# Microbiome or no microbiome: are we looking at the prenatal environment through the right lens?

**DOI:** 10.1186/s40168-020-00947-1

**Published:** 2021-01-12

**Authors:** W. Florian Fricke, Jacques Ravel

**Affiliations:** 1 Department of Microbiome Research and Applied Bioinformatics, University of Hohenheim, Stuttgart, Germany; 2 Institute for Genome Sciences and Department of Microbiology & Immunology, University of Maryland School of Medicine, MD Baltimore, USA

Over the past two decades, technological advances, primarily those in DNA sequencing, have spearheaded a revolution in human microbiome research. As a consequence, the field has made major strides forward and clearly established a role for the human microbiome in health and disease. The accessibility, ease of use, and low cost of 16S rRNA gene amplicon sequencing-based approaches have led to their application to all kinds of samples, some of which collected from body sites originally thought to be sterile including the brain, the placenta or even the growing fetus. The sensitivity and phylogenetic resolution afforded by these methods resulted in the detection of DNA fragments specific to bacteria at these sites. Clinical paradigms were challenged by considering evidence of bacteria in these human specimens not as an unmistakable sign of infection and going to such lengths as postulating microbiota colonization as a prerequisite of health. These early reports stimulated further enthusiasm for such human microbiome studies but were not universally well-received and have drawn criticisms for their lack of careful experimental, technical, bioinformatic and statistical controls and sometimes rigor. A controversy started.

In 2014 Aagaard et al. published a study that reported on the presence of a bacterial microbiota in placental specimens collected from healthy pregnant women after delivery [1]. The paper created broad excitement [2], but was also met with skepticism by infectious disease specialists and others [3], as it challenged the established thinking on the topic stating that bacteria in placental tissue represent a sign of infection. The existence of a “healthy” placental microbiota begged the question of in utero bacterial colonization of the growing fetus, i.e., whether there is a prenatal microbiome. A recent contribution to this field of research by Rackaityte et al. (2020), while not the first report, provided multiple lines of evidence for the presence of bacterial DNA and cellular structures in fetal intestines, as well as cultivable *Micrococcus luteus*-related bacteria that appeared to show adaptations to the fetal environment, including growth on pregnancy hormones and survival within phagocytic cells [4]. However, key findings from this study were criticized in a recent letter to the editor submitted to *Microbiome* by De Goffau et al. (2020). After long discussions with the Editorial Board of *Microbiome*, it was decided that De Goffau’s claims and data appeared convincing but that the letter should be peer-reviewed. A revised version of the letter was accepted for publication in *Microbiome* [5]. This letter, together with a response from the authors of the original publication [6], appears in this special issue of *Microbiome*.

As the editorial board considered the letter and the original authors’ response, it was felt that the questions and concerns raised by De Goffau et al. carried broader implications not only for the field of prenatal microbiome research but also for scientists studying other body sites previously thought to be sterile and with known low biomass, as well as for the microbiome field in general. Several experts were asked to provide their assessment and thoughts on the prenatal microbiome and the lessons learned for the field, as well as to reflect on the communication of science. In order to broaden the perspective, scientists were selected who have a long-standing expertise in microbiome sciences but have not been directly involved in the debate. To provide some context and a broader view of the topic to our readership, two commentaries are also included into the special issue that reflect different perspectives on the prenatal and fetal microbiomes (Walter & Hornef [7] and Silverstein & Mysorekar [8]).

The prenatal microbiome controversy and the experts’ perspectives offer important insights into some of the broader challenges and limitations of current microbiome research. As a common technical issue, contamination of biological samples with trace amounts of bacterial DNA from sampling sites, clinical or laboratory environments, as well as reagents and consumables, is widely recognized as a source of false positive microbiome signals, particularly for many low-biomass specimen types [9]. Other sources of 16S rRNA amplicon artifacts, such as index or barcode crosstalk, well-to-well contamination, or non-target amplification have been described [10, 11] and are important but less well characterized. It is widely accepted but rarely implemented that the inclusion of proper process controls as well as biological and technical replicates should be a fundamental part of the experimental design for any microbiome study, and more specifically those dealing with low biomass or previously thought sterile sample types. A stricter enforcement of open data and methods sharing (laboratory and informatics) is absolutely necessary to increase rigor and transparency, and ideally allow for retrospective checks on published data. We will continue to promote and enforce their use in publications published in *Microbiome*.

A more conceptual problem of many microbiome studies is their application of an almost forensic approach to detect and define microbiomes, solely based on the amplification of bacterial DNA signals. This is problematic in several ways. The presence of bacterial DNA alone is certainly not sufficient to infer the presence of a thriving microbial community, e.g. in the placenta, which would be in itself an argument to refute the prenatal microbiota hypothesis. Further, if biological activity is required to confirm a physiological role, it would not have to be limited to live microbes, as bacterial DNA or microbial cell wall components could also invoke a host response. Thus, with functional assays that measure microbiome relevance based on host response, not just presence of microbes or their DNA, one could study the microbiome through the lens of the host and advance our understanding of the microbiome’s role for human health. Lastly, proof of microbiome presence should not be considered as an end in itself, but health benefits to the infant need to be established. Long term clinical studies are desperately needed that would combine compositional and functional examination of the microbiome in relation to broad measures of infant growth and health.

Microbiome is committed to support all areas of microbiome research, including those investigating potential host-microbe interactions at prenatal stages. The editorial board strongly believes that functional and mechanistic studies that test hypotheses on specific interactions between microbes and the human host, and their role in health or disease, have the potential to substantially improve our understanding of the human microbiome. Such studies will lead to innovative ways to manipulate these microbiomes and improve health. *Microbiome* is further committed to continue promoting an open discussion of existing methods and goals of microbiome research, including challenging established paradigms. We strongly believe in the importance of open data and protocols to foster these critical discussions. The reanalysis by De Goffau et al. of the original data from Rackaityte et al. is an example of the kind of transparent research communication *Microbiome* will continue to promote.
